# Supplementary material for: Seed type, habitat and time of day influence post-dispersal seed removal in temperate ecosystems
Source: PeerJ. 2020 Mar 13;8:e8769. doi: 10.7717/peerj.8769 (PMC7075361; doi:10.7717/peerj.8769)
Supplement: Supplemental Information 1 — LoCo, Lotus corniculatus; ChMa, Chelidonium majus; dhHeAn, dehulled Helianthus annuus; inHeAn, intact Helianthus annuus. [file peerj-08-8769-s001.pdf]

Appendix 1. GPS coordinates of forest and grassland sites in Darmstadt, Airlenbach and Zell in South Hesse, Germany. LoCo = *Lotus corniculatus* , ChMa = *Chelidonium majus* , hHeAn = hulled *Helianthus annuus* , uHeAn = unhulled *Helianthus annuus* .

**plot_ID**	**north**	**east**	**habitat**	**region**	**seed type**	**daytime**
Post-dispersal seed removal and seed type						
1	49.857550	8.678983	forest	Darmstadt	all seed types	day
2	49.857750	8.677800	grassland	Darmstadt	all seed types	day
3	49.856717	8.687950	forest	Darmstadt	all seed types	day
4	49.859467	8.686967	grassland	Darmstadt	all seed types	day
5	49.589933	8.926767	forest	Airlenbach	all seed types	day
6	49.589450	8.925950	grassland	Airlenbach	all seed types	day
Post-dispersal seed removal and daytime						
41	49.86560	8.67948	forest	Darmstadt	LoCo/ChMa/hHeAn/uHeAn	day/night
42	49.86753	8.68029	grassland	Darmstadt	LoCo/ChMa/hHeAn/uHeAn	day/night
43	49.86686	8.68013	forest	Darmstadt	LoCo/ChMa/hHeAn/uHeAn	day/night
44	49.86103	8.68619	grassland	Darmstadt	LoCo/ChMa/hHeAn/uHeAn	day/night
45	49.86121	8.68788	forest	Darmstadt	LoCo/ChMa/hHeAn/uHeAn	day/night
46	49.85990	8.68636	grassland	Darmstadt	LoCo/ChMa/hHeAn/uHeAn	day/night
47	49.85693	8.68744	forest	Darmstadt	LoCo/ChMa/hHeAn/uHeAn	day/night
48	49.85720	8.68580	forest	Darmstadt	LoCo/ChMa/hHeAn/uHeAn	day/night
49	49.85783	8.67902	forest	Darmstadt	LoCo/ChMa/hHeAn/uHeAn	day/night
50	49.85846	8.67818	grassland	Darmstadt	LoCo/ChMa/hHeAn/uHeAn	day/night
51	49.85885	8.67884	grassland	Darmstadt	LoCo/ChMa/hHeAn/uHeAn	day/night
52	49.86560	8.67936	grassland	Darmstadt	LoCo/ChMa/hHeAn/uHeAn	day/night
53	49.59454	8.92322	grassland	Airlenbach	LoCo/ChMa/hHeAn/uHeAn	day/night
54	49.60108	8.92363	forest	Airlenbach	LoCo/ChMa/hHeAn/uHeAn	day/night
55	49.60038	8.92011	grassland	Airlenbach	LoCo/ChMa/hHeAn/uHeAn	day/night
56	49.60119	8.91945	grassland	Airlenbach	LoCo/ChMa/hHeAn/uHeAn	day/night
57	49.60140	8.91940	forest	Airlenbach	LoCo/ChMa/hHeAn/uHeAn	day/night
58	49.60119	8.91945	forest	Airlenbach	LoCo/ChMa/hHeAn/uHeAn	day/night
59	49.59946	8.91281	grassland	Airlenbach	LoCo/ChMa/hHeAn/uHeAn	day/night
60	49.59882	8.90799	forest	Airlenbach	LoCo/ChMa/hHeAn/uHeAn	day/night
61	49.59827	8.90898	forest	Airlenbach	LoCo/ChMa/hHeAn/uHeAn	day/night
62	49.59771	8.90854	grassland	Airlenbach	LoCo/ChMa/hHeAn/uHeAn	day/night
63	49.59770	8.91023	grassland	Airlenbach	LoCo/ChMa/hHeAn/uHeAn	day/night
64	49.59000	8.92646	forest	Airlenbach	LoCo/ChMa/hHeAn/uHeAn	day/night
65	49.71755	8.99042	grassland	Zell	LoCo/ChMa/hHeAn/uHeAn	day/night
66	49.71172	8.99162	grassland	Zell	LoCo/ChMa/hHeAn/uHeAn	day/night
67	49.71300	8.99037	grassland	Zell	LoCo/ChMa/hHeAn/uHeAn	day/night
68	49.71230	8.99036	grassland	Zell	LoCo/ChMa/hHeAn/uHeAn	day/night
69	49.71342	8.99003	forest	Zell	LoCo/ChMa/hHeAn/uHeAn	day/night
70	49.71118	8.98912	forest	Zell	LoCo/ChMa/hHeAn/uHeAn	day/night
71	49.71032	8.98934	forest	Zell	LoCo/ChMa/hHeAn/uHeAn	day/night
72	49.70643	8.99390	forest	Zell	LoCo/ChMa/hHeAn/uHeAn	day/night
